# Effects of Bergamot Polyphenols on Mitochondrial Dysfunction and Sarcoplasmic Reticulum Stress in Diabetic Cardiomyopathy

**DOI:** 10.3390/nu13072476

**Published:** 2021-07-20

**Authors:** Jessica Maiuolo, Cristina Carresi, Micaela Gliozzi, Vincenzo Musolino, Federica Scarano, Anna Rita Coppoletta, Lorenza Guarnieri, Saverio Nucera, Miriam Scicchitano, Francesca Bosco, Stefano Ruga, Maria Caterina Zito, Roberta Macri, Antonio Cardamone, Maria Serra, Rocco Mollace, Annamaria Tavernese, Vincenzo Mollace

**Affiliations:** 1 IRC-FSH Department of Health Sciences, University “Magna Græcia” of Catanzaro, Campus Universitario di Germaneto, 88100 Catanzaro, Italy; jessicamaiuolo@virgilio.it (J.M.); carresi@unicz.it (C.C.); micaela.gliozzi@gmail.com (M.G.); xabaras3@hotmail.com (V.M.); federicascar87@gmail.com (F.S.); annarita.coppoletta@libero.it (A.R.C.); lorenzacz808@gmail.com (L.G.); saverio.nucera@hotmail.it (S.N.); miriam.scicchitano@hotmail.it (M.S.); fra.bosco88@gmail.com (F.B.); rugast1@gmail.com (S.R.); mariacaterina.zito@gmail.com (M.C.Z.); robertamacri85@gmail.com (R.M.); antonio.cardamone@studenti.unicz.it (A.C.); maria.serra@gmail.com (M.S.); rocco.mollace@gmail.com (R.M.); an.tavernese@gmail.com (A.T.); 2 Nutramed S.c.a.r.l, Complesso Ninì Barbieri, Roccelletta di Borgia, 88021 Catanzaro, Italy; 3 IRCCS San Raffaele, Via di Valcannuta 247, 00133 Rome, Italy

**Keywords:** diabetic cardiomyopathy, myocardial metabolism, myocardial dysfunction, role of mitochondria and sarcoplasmic reticulum in the myocardium, bergamot polyphenols, bergamot polyphenolic fraction

## Abstract

Cardiovascular disease is the leading cause of death and disability in the Western world. In order to safeguard the structure and the functionality of the myocardium, it is extremely important to adequately support the cardiomyocytes. Two cellular organelles of cardiomyocytes are essential for cell survival and to ensure proper functioning of the myocardium: mitochondria and the sarcoplasmic reticulum. Mitochondria are responsible for the energy metabolism of the myocardium, and regulate the processes that can lead to cell death. The sarcoplasmic reticulum preserves the physiological concentration of the calcium ion, and triggers processes to protect the structural and functional integrity of the proteins. The alterations of these organelles can damage myocardial functioning. A proper nutritional balance regarding the intake of macronutrients and micronutrients leads to a significant improvement in the symptoms and consequences of heart disease. In particular, the Mediterranean diet, characterized by a high consumption of plant-based foods, small quantities of red meat, and high quantities of olive oil, reduces and improves the pathological condition of patients with heart failure. In addition, nutritional support and nutraceutical supplementation in patients who develop heart failure can contribute to the protection of the failing myocardium. Since polyphenols have numerous beneficial properties, including anti-inflammatory and antioxidant properties, this review gathers what is known about the beneficial effects of polyphenol-rich bergamot fruit on the cardiovascular system. In particular, the role of bergamot polyphenols in mitochondrial and sarcoplasmic dysfunctions in diabetic cardiomyopathy is reported.

## 1. Introduction

Cardiovascular diseases represent the main cause of death and disability in the Western world; in recent decades, there has been an increase in disorders caused by heart failure (HF). Several risk factors [1,2,3,4,5] contribute to the onset of HF that can be distinguished into non-modifiable, (sex, age, family predisposition, genetic factors, aging) [6], partially modifiable, (obesity, diabetes mellitus, hypercholesterolemia, arterial hypertension, increase in radical species, increase in systemic inflammatory processes) [7,8,9], and modifiable (alcohol and drug abuse, physical inactivity, high-calorie diet rich in saturated fat and salt) [10,11,12,13,14]. In addition, HF often occurs in patients with comorbidities such as diabetes, hypertension, and dyslipidemia [15].

**Figure 1:**
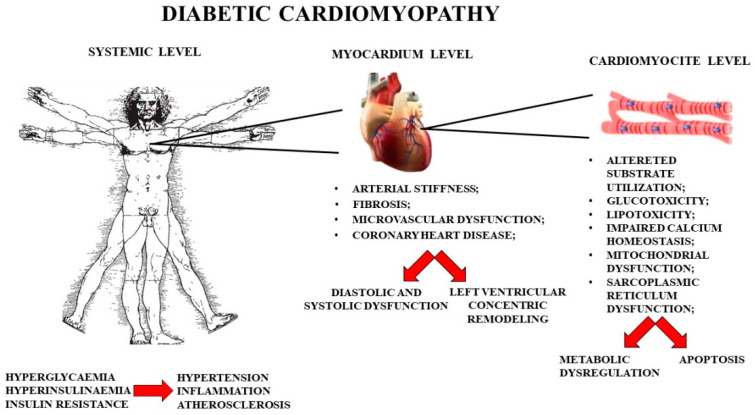
Characteristics of systemic, organ (myocardium), and cellular (cardiomyocytes) diabetic cardiomyopathy.

Diabetic cardiomyopathy is generated by insulin resistance, hyperinsulinemia, and hyperglycemia. These metabolic changes can occur independently of other heart disorders, including coronary artery disease and hypertension [16]. In fact, the risk of developing heart failure is increased in diabetic patients (about 2.4 times in men and 5 times in women) compared with those without diabetes. Heart disease, linked to diabetes, is responsible for structural and functional adaptations that sequentially occur. In the early stages of diabetic cardiomyopathy, frequent pathophysiological changes occur, such as the impaired autophagy of cardiomyocytes, the increased death of cardiomyocytes, the inappropriate activation of the renin–angiotensin–aldosterone system, oxidative stress, and maladaptive immune responses, responsible for the onset of fibrosis and substantial cardiac stiffness. In addition, metabolic disorders such as reduced insulin signaling, decreased glucose uptake, increased myocardial absorption of non-esterified fatty acids, and mitochondrial dysfunction are responsible for heart remodeling, fibrotic diastolic dysfunction, and decreased ejection fraction [17,18]. In later stages of diabetic cardiomyopathy, changes in myocardial structure are more pronounced and include necrosis, increased connective-tissue cross-linking, interstitial fibrosis, collagen accumulation, hypertrophy, and capillary microaneurysms [19]. Further changes are associated with coronary microcirculation, and diastolic and systolic dysfunction [20]. Hyperglycemia and glucotoxicity emphasize multiple instances of cell damage, including the glycation of biological macromolecules such as proteins and lipids. In particular, the deposition of advanced glycation end products contributes to the increase in connective-tissue cross-linking and fibrosis, worsening diastolic relaxation and cardiac stiffness. Lastly, these compounds are involved in an increase in reactive oxygen species (ROS) and inflammatory processes [21]. Impaired metabolic insulin signaling is also associated with diabetic cardiomyopathy. Under physiological conditions, cardiomyocytes, via the PI3K/Akt signaling pathway, stimulate the recruitment of glucose carrier GLUT4 to the plasma membranes, which results in glucose uptake. When insulin resistance is activated, PI3K/Akt pathway is altered, resulting in a reduction in glucose in the heart [22]. A poor diet, rich in refined fats and carbohydrates, and physical inactivity contribute to the reduced metabolic signaling of cardiac insulin [23,24]. The mammalian heart requires a high level of energy, and cellular mitochondria are essential to ensure this function, as they generate over 95% of the ATP used by the heart. Therefore, the proper metabolism of cardiomyocytes is responsible for the optimal functioning of the myocardium [25]. Furthermore, cardiomyocytes also regulate cell signaling and death, and the availability of the calcium ion, responsible for the muscle-contraction mechanism; cardiomyocyte mitochondria also regulate cell signaling and death. The extent of muscle contraction depends on the availability of calcium ion levels. The sarcoplasmic reticulum, together with the mitochondria, is able to regulate the homeostasis of intracellular calcium, essential for the process of the excitation–contraction of the heart [26]. Therefore, the proper functioning of the mitochondria, the sarcoplasmic reticulum, and their appropriate cross-talk is the basis for the functioning of the cardiomyocytes and the myocardium [27]. The onset of diabetic cardiomyopathy at the systemic, organ, and cellular levels is represented in Figure 1.

To date, there have been many studies showing that proper nutrition could be an important factor in the prevention of HF [28]. Food is the primary source of energy, essential to support a proper myocardial contractility [6], and macro- and micronutrients must be perfectly balanced in the diet [29,30]. Current scientific knowledge shows that a high-fat diet is associated with heart failure and arrhythmias [31,32]. For example, a recent in vivo study was conducted on male mice fed with a high-fat diet for 28 weeks [33]. At the end of the experimental period, cardiac parameters, myocardial lipid content, and mitochondrial function and morphology were evaluated. The results obtained showed a significant increase in myocardial fat, marked cardiac hypertrophy, and morphological abnormalities of the mitochondrial structure in mice treated with a high-fat diet compared to the control group fed a normal diet. In addition, in the same experimental group, the abnormal expression of genes involved in mitochondrial dynamics, a reduction in the expression of mitochondrial respiration protein complexes, and a significant increase in the AMP/ATP ratio were observed [33]. Therefore, greater adherence to a healthy diet significantly reduces the risk of developing cardiovascular diseases [34,35]. In this context, the Mediterranean diet, consisting mainly of fruit, vegetables, whole grains, nuts, seeds, and legumes can reduce the excessive production of proinflammatory cytokines and the accumulation of ROS [36,37,38,39].

Bergamot (*Citrus bergamia*, Risso et Poiteu), is a citrus fruit that grows almost exclusively in southern Italy, in a restricted area of the Calabrian coast; it is characterized by a unique profile in flavonoid glycosides, including neoeriocitrin, neohesperidin, naringin, and glycosylated polyphenols, such as bruteridin and melitidin [40]. Bergamot was initially grown for its use in the perfumery, cosmetic, food, and confectionery industries, but is now known to possess several pharmacologically beneficial effects on various organs and tissue types. To date, the protective effects of the main polyphenols contained in high concentration in bergamot are well-known. In particular, important beneficial effects on the nervous system [41,42,43], the mineralization of bones [44], the regulation of keratinocytes [45], the regulation of the metabolism [46,47], against adipose-tissue inflammation [48] and during myocardial regeneration [49] were reported. These protective effects of bergamot have been found in many of its formulations: extract (BE), juice (BJ), essential oil (BEO), and polyphenolic fraction (BPF). In particular, although there are not many studies carried out on animal models concerning the protective effect of bergamot in muocardium, it is known that the treatment with BPF (10 or 20 mg/kg/daily for 30 days) reduces the total cholesterol, LDL, and triglycerides, enhancing fecal sterols excretion, compared to controls and this treatment does not generate toxicity related to liver detoxication [50]. The studies on humans reported a cardio-protective effect of a single daily dose of BE (150 mg of flavonoids), with 37% of naringin, 47% of neohesperidin and 16% of neoeriocitrin for 6 months [51]. Other studies agree that the administration of BPF per 30 days, at a daily dose of 500 mg or 1000 mg, is able to support heart health in humans and again these dosages do not interfere with liver detoxification metabolism [52,53].

In particular BPF, an enriched polyphenolic formulation obtained from the juice and albedo of bergamot, has protective activities in the management of atherosclerosis, metabolic disorders, and cardiotoxicity due to its antioxidant, anti-inflammatory, and lipid-lowering effects [54,55,56,57]. Furthermore, BPF reduces serum cholesterol and triglyceride levels, improving systemic inflammation and endothelial function [58,59,60,61]. Interesting experimental data also demonstrate the beneficial protective action of BPF in preventing hyperglycemia [62,63].

Therefore, the present review reports two important topics in depth:

- The functional role of mitochondria and sarcoplasmic reticulum in cardiomyocytes in physiological conditions and in the onset of diabetic cardiomyopathy.
- The potential beneficial role of bergamot polyphenols in diabetic cardiomyopathy.

### 1.1. Myocardial Metabolism

Cardiomyopathies are pathological conditions consisting of myocardial alterations that often lead to heart failure. The American Heart Association distinguishes primary and secondary cardiomyopathies: the former are disorders that only affect the heart, while the latter describe conditions in which the cardiac alteration is accompanied by dysfunctions in other bodily districts [64,65]. Although this distinction is currently recognized, the symptoms of one form can often overlap with those of another [66,67].

The first fundamental aspect that should be addressed, which concerns myocardial cells, is the available energy sources in order to ensure excitation–contraction. Cardiomyocytes are capable of utilizing all classes of energy substrate to produce ATP, including carbohydrates, lipids, amino acids, and ketone bodies. Of the energy needed by myocardial cells to ensure muscle contraction, 70% is obtained from the oxidation of fatty acids. The absorption of circulating fatty acids occurs in cardiomyocytes by passive diffusion or facilitated transport by the translocation enzyme of fatty acids located near the plasma membrane [68]. However, the transport of fatty acids into the mitochondria is quite different. In this case, support is required for a specific translocation, a protein that binds to fatty acids and transports them inside the mitochondria [69]. The entry of acyl-CoA into the mitochondria is a finely regulated process carried out by the carnitine-palmitoyl-transferase I (mCPT-1) enzyme, present on the outer portion of the inner mitochondrial membrane, which catalyzes the transfer of acyl-CoA to carnitine [70]. The action of mCPT-1 is the phase that determines the level of entry of fatty acids into the mitochondria, and enzymatic activity is allosterically regulated by the malonyl-CoA inhibitor [71]. In response to low levels of chemical energy, a regulated reduction in malonyl-CoA levels and increased activity of the mCPT-1 enzyme are triggered. Once in position, fatty acids are degraded in the Krebs cycle generating FADH2 and NADH, which are the reducing equivalents that feed the electron transport chain resulting in the consumption of oxygen and the final formation of ATP. Although fatty acids are the main source of energy, cardiomyocytes have low storage capacity [72]. In addition, under conditions of low oxygen concentrations, energy is derived from the cleavage of other substrates such as glucose, lactate, and ketone bodies. The oxidation of pyruvate is regulated by the pyruvate dehydrogenase enzyme. Ketone metabolism produces acetyl-Coa, while the catabolism of amino acids produces ketoacids that are further metabolized to enter the Krebs cycle [73]. The degradation of glucose and lactate by glycolysis and dehydrogenation, respectively, ensure an amount of energy equal to 10–30% of cardiomyocyte needs and, although the production of ATP is greater by the oxidation of fatty acids, the degradation of glucose and lactate is more effective because it consumes fewer oxygen molecules [74]. The transport of glucose inside the cardiomyocytes is facilitated by the glucose transporters (Gluts) present on the membranes of the sarcolemma. The most expressed isoforms are GLUT1, constitutively active, and GLUT4, which results in its expression in the sarcolemma in response to insulin production. In general, enzymes involved in the oxidative metabolism of fatty acids or the degradation of glucose are finely regulated at the transcriptional level [75]. In light of the foregoing regarding myocardial metabolism, the need for cardiomyocytes to possess a large number of mitochondrial organelles to satisfy the demand for large amounts of energy is justified. For this reason, cardiomyocytes are the cells with the highest number of mitochondria, and it is estimated that these organelles occupy about one-third of the cell volume of the cardiomyocyte. Mitochondrial dysfunction, which occurs in pathological conditions, reduces the oxidative metabolism by generating changes in the myocardium [76,77]. Several scientific studies showed that there is a link between the altered metabolism of cardiac energy substrates and the accumulation of ROS [78,79].

### 1.2. Role of Calcium Ion in the Myocardium

The calcium ion is a key element in all eukaryotic cells, as it is involved in many signaling processes such as differentiation, proliferation, apoptosis, excitation, cell contraction, and neuronal plasticity [80]. In the myocardium, calcium is involved in both electrical activity and cardiac contractility [81], and also influences the regulation of gene expression; this process is excitation–transcription coupling [82]. The direct consequence is that there are forms of the strict regulation of calcium ion in order to maintain its correct homeostasis. In particular, calcium transport mechanisms are present between the cell cytosol and the extracellular environment, and between the cell cytosol and calcium deposits maintained in the sarcoplasmic reticulum. The increase in the cellular availability of calcium occurs mainly, but not exclusively, as a result of the release of this ion from the sarcoplasmic reticulum. Calcium determines conformational changes in the troponin–tropomyosin complex, the sliding of the actin and myosin filaments, and muscle contraction. Conversely, sarcomere relaxation occurs as a result of cytosolic calcium reduction, the reuptake of the sarcoplasmic reticulum through the SERCA pump, and/or by the Na^+^/Ca^2+^ exchanger [83]. In cardiomyocytes, changes in calcium ion homeostasis are related to many forms of heart failure, and dysfunctional ion transport from the sarcoplasmic reticulum was found in the pathophysiology of heart failure [84]. Diabetic cardiomyopathy is associated with a reduced reuptake of Ca^2+^, which causes an increase in the potential of action and a slowdown in diastolic relaxation. These effects are responsible for inducing the apoptotic and necrotic death of cardiomyocytes. Furthermore, autophagic impairment, which occurs with alterations in autophagosomal and lysosomal fusion, is also involved in diabetic cardiomyopathy [85].

### 1.3. Importance of Mitochondria and the Sarcoplasmic Reticulum in the Myocardium

The mitochondrial dysfunction of cardiomyocytes is related to the onset of myocardial damage, which can evolve in the development of numerous diseases [86]. Any form of mitochondrial insult is able to alter the shape and functions of these organelles, and the membrane potential and its metabolism. In physiological conditions, mitochondria carry out oxidative phosphorylation, producing ROS, which do not, however, cause damage to myocardial cells due to the presence of endogenous antioxidant mechanisms that can counteract its accumulation [87]. If an insult affecting myocardial cells reduces the levels of endogenous antioxidants, the balance between pro- and antioxidant mechanisms is disrupted, and ROS can interact and alter biological macromolecules such as proteins, nucleic acids, and lipids. In particular, ROS can modify the structure and function of proteins, alter DNA repair mechanisms, and generate lipid peroxidation, interfering with biological membranes [88,89]. There is a complex mechanism in the cell, mitochondrial quality control (MQC), which removes or repairs damaged mitochondria, and maintains proper mitochondrial morphology, quantity, and function [90]. In particular, MQC is able to implement post-translational modifications of mitochondrial proteins to collaborate in mitochondrial biogenesis through mechanisms that lead to the fission and fusion of mitochondria, and to trigger mitochondrial autophagy (mitophagy), leading to an irreversible degradation in lysosomes when necessary [91,92]. Mitochondrial dynamics (including their biogenesis, number, and shape) comprise several processes, such as fusion, fission, and mitophagy [93]. Among factors responsible for the implementation of correct mitochondrial dynamics, one of the most important is the transcriptional coregulator peroxisome proliferator-activated receptor γ (PPAR γ), coactivator 1α (PGC1α), which promotes the transcription of numerous genes responsible for mitochondrial biogenesis. This protein is expressed in all energy-consuming tissue types, including striated and skeletal muscle, brown fat, the liver, and the brain [94]. Studies in the recent scientific literature showed that the overexpression of PGC1α causes the accumulation of enlarged mitochondria in vitro and the loss of sarcomeric structure with dilated cardiomyopathy in vivo [95]. Conversely, the lack of PGC1α was not associated with abnormal mitochondrial and cardiac structure, although it resulted in the deficient expression of mitochondrial proteins and contractile cardiac dysfunction in vivo [96]. In view of the above, certain alterations in mitochondrial dynamics can be determined under pathological conditions (direct damage or exposure to cardiotoxic agents), leading to mitophagy to save cellular homeostasis [97]. In addition to biological macromolecules, these reactive species also damage mitochondrial DNA (mtDNA). Under these circumstances, mutations and deletions accumulate, eventually leading to the development of dilated cardiomyopathy and an aging-like condition [98]; for example, following ultrastructural analysis, myocardial mitochondria appeared to be enlarged and swollen, with no crests, with a damaged matrix, and low levels of ATP production. When mitophagy fails to eliminate damaged mitochondria, cardiomyocytes undergo apoptosis [99]. In diabetic cardiomyopathy, hyperglycemia induces the oxidation of intracellular glucose, generating an increase in pyruvate and its influx into the mitochondria. The consequence is an elevated production of ROS, generated at complexes I and III of the mitochondrial chain, a reduction in oxidative phosphorylation, and the generation of mitochondrial ATP [100]. In addition, the increased absorption of mitochondrial fatty acids exceeds mitochondrial respiration capacity, and induces an accumulation of toxic lipid metabolites that cause mitochondrial dysfunction and cardiac lipotoxicity [101].

The heart needs calcium to function properly, and the main source of this ion is the sarcoplasmic reticulum (SR). The release of calcium from SR is modulated by a set of proteins, and particularly by the ryanodine receptor (RyRs). RyRs form a class of intracellular calcium channels found in mammals, and are excitable in muscle and nerve cells. Their role is to mediate the release of calcium ions from the sarcoplasmic reticulum so that they can perform muscle contraction. There are three known isoforms of RyRs, and RyR2 is the isoform expressed mainly in the myocardium, where it is responsible for the excitation–contraction coupling process (E–C coupling). Mutations in RyR2 are associated with changes in the heartbeat [102]. RyRs are very close to mitochondria, and the release of calcium from RyR2 regulates the production of ATP in cardiomyocytes [103]. These channels regulate the release of calcium ions via negative feedback modulated by cytosolic calcium concentrations [104]. Two small molecules capable of modulating RyRs are Mg^2+^ and ATP; the Mg^2+^ ion inhibits these channels with two mechanisms:

- Reducing the opening of RyRs by competing with Ca^2+^ sites with higher affinity;
- Reducing the opening of RyRs by binding to less selective sites for Ca^2+^ [105].

Some RyRs can be activated even in the absence of Ca^2+^ [103]. ATP, ADP, AMP, cAMP and adenosine can also activate RyRs, but ATP is more effective [104].

Another alteration involving the sarcoplasmic reticulum of cardiomyocytes is the imbalance of the proteostasis network, which normally ensures the integrity of proteins. Proteostasis includes the regulation of synthesis, folding, trafficking, and degradation.

The correct structure and functioning of proteins are essential to ensure cardiac contraction: among the main ones are some ionic channels, proteins associated with the viability of cardiomyocytes, growth factors, hormones, and proteins responsible for interaction with other cell types [105]. An imbalance between the components of these networks can lead to the accumulation of misfolded proteins, proteinopathy, or proteotoxicity. These alterations in cardiomyocytes can lead to ischemic phenomena and hypertrophic or dilated cardiomyopathies [106]. An excess of misfolded proteins results in the production of toxic polypeptides that can affect myocardial function and lead to heart failure [107]. In general, three mechanisms through which cells are able to remove potentially toxic misfolded proteins are recognized:

- Degradation through the ubiquitin proteasome system (UPS); UPS components are located in specific regions of cells. For example, in cardiomyocytes UPS, elements are found on the Z line of sarcomeres, in the cytoplasm, in the nucleus, and on the surface of many organelles, such as SR, mitochondria, and lysosomes [108].
- A proteosome-independent process involving autophagy in different districts; this activates mitophagy [109] or SR autophagy [110].
- A physiological response following the increase in unfolded proteins in the SR, which activates the unfolded protein response (UPR) [111]. The ATF6 branch of UPR is involved in many cellular processes, including a rearrangement of lipid synthesis with the aim of reducing damage due to the accumulation of unfolded proteins [112]. The SR involves ATF6 in protein misfolding, cardiomyopathy, and heart failure [113]. ATF6 also participates in the induction of those genes that reprogram proteostasis, reducing the death of cardiomyocytes and conferring cardioprotection [114].

**Figure 2:**
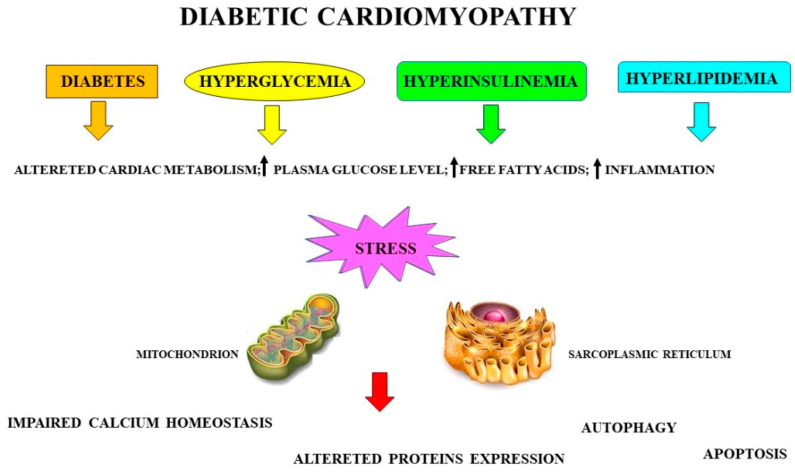
Involvement of mitochondria and sarcoplasmic reticulum in stress induced by diabetic cardiomyopathy, showing how diabetes, hyperglycemia, hyperinsulinemia, and hyperlipidemia cause a stress condition involving dysfunction of the mitochondria and sarcoplasmic reticulum. In particular, some events occur including an impaired calcium homeostasis, altered protein expression, autophagy, and apoptotic cell death.

In diabetic cardiomyopathy, the myocardium could be damaged as the main consequence of hyperglycemia, hyperinsulinemia, and hyperlipidemia. A summary is shown in Figure 2.

### 1.4. Sarcoplasmic-Reticulum Stress in Diabetic Cardiomyopathy

**Figure 3:**
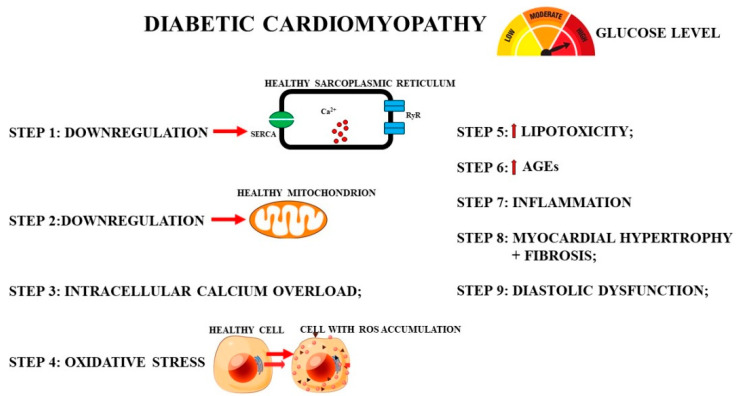
Steps involved in diabetic cardiomyopathy in temporal order. (1) Downregulation of SERCA pump in the sarcoplasmic reticulum; (2) mitochondrion downregulation; (3) consequent increase in cytosolic calcium; (4) accumulation of oxidative stress in cells; (5) increase in lipotoxicity; (6) increase in advanced glycation products; (7) inflammation; (8) rearrangement of myocardial tissue with hypertrophy and fibrosis; (9) onset of diastolic dysfunction.

In the last decade, a correlation was found between SR stress and diabetic cardiomyopathy [101,115,116]. Pathological remodeling in this disease is accompanied by the alteration of cellular proteins that can facilitate SR stress, and alterations in the intracellular homeostasis of Ca^2+^ and UPR [117]. The factors that induce reticulum stress could be hyperglycemia, free fatty acid accumulation, and insulin deficiency or resistance and inflammation [118]: for this reason, SR stress is an early event in diabetic cardiomyopathy [119]. In particular, hyperglycemia alters SR homeostasis, and glucose, normally used as energy fuel, is metabolized to generate harmful compounds. Damage that can lead to cardiomyocyte death occurs when a high glucose overload is also accompanied by lipid accumulation, inducing oxidative stress responsible for the dysregulation of protein homeostasis [78,120]. In these circumstances, the UPR is involved: in the presence of glucose overload alone, the PERK and ATF6 arms of UPR are activated [121]; in the case of both glucose and lipid accumulation, signaling involving IRE-XBP1, the third arm of UPR, is upregulated [115]. In light of clinical evidence, the UPR of SR can demonstrate both adaptive and maladaptive roles in the heart, and a prolonged or dysfunctional UPR can lead to heart disease. In particular, an increase in CCAAT-enhancer-binding protein homologous protein (CHOP) expression leads to cell death. CHOP is a transcription factor whose expression is low under normal conditions but increases under SR stress. Overexpression of CHOP promotes apoptosis in cardiomyocytes [122,123]. In diabetic cardiomyopathy, prolonged SR stress determines the increase in the expression of CHOP and c-Jun N-terminal kinases (JNK) responsive to stress stimuli, and caspase 12 activation [124]. Furthermore, the accumulation of saturated fatty acids inhibits SERCA channel activity due to the loss of membrane fluidity, causing an alteration of Ca^2+^ and UPR, and triggering early lipotoxic heart stress [125]. A recent scientific study highlighted the involvement of the SERCA pump due high glucose concentrations [126]. Since there is cross-talk between calcium homeostasis and the redox state in cells, hyperglycemia also alters oxidative homeostasis in mitochondria [78,127]. This is the reason for the progressive accumulation of ROS and reactive nitrogen species, advanced glycation, organelle dysfunction, and chronic inflammation in diabetic cardiomyopathy [128]. Hyperinsulinemia also appears to be involved in SR stress via two mechanisms [129,130]:

- The IRE1/JNK signaling pathway is activated, contributing to a further reduction in cardiac function [131];
- The onset of an inflammatory state: during diabetic cardiomyopathy, it induces the activation of macrophages, neutrophils, mast cells, platelets, and T lymphocytes, leading to the release of proinflammatory cytokines and other molecules, such as ROS and proteases, which have harmful effects on cardiomyocytes [132]. The induced inflammatory response contributes to the onset and development of cardiomyopathy and heart failure [133]. Figure 3 shows the phases that temporally occur during the onset of myocardial damage caused by hyperglycemia in diabetic cardiomyopathy.

## 2. Diabetic Cardiomyopathy and Bergamot Polyphenols

Growing evidence suggests that a control of nutritional balance (with particular regard to the intake of micronutrients and nutraceuticals), in patients with heart disease leads to a significant improvement in symptoms and outcomes [134,135]. Cardiomyocytes obtain about 70% of the required energy by the mitochondrial β-oxidation of fatty acids; the excess part is esterified and stored in the form of energy reserves in lipid drops in the cell cytoplasm. If the diet is based on excessive fat consumption, however, fatty acids accumulate in the myocardium and around the heart; this can also generate deleterious effects on blood circulation, since toxic metabolic derivatives such as ROS and ceramides can occur, causing lipotoxicity phenomena, leading to severe dysfunctions in cardiomyocytes [136,137]. In particular, numerous experimental in vitro and in vivo studies highlighted how a high-fat diet is responsible for heart failure, myocardial hypertrophy, and myocardial lipid accumulation [138]. A high-fat diet during gestation in several species causes deleterious effects in newborns; in particular, altered gene expression, abnormalities in the functions of antioxidant enzymes, the increased possibility of developing atherogenesis, and damage to the cardiovascular system [139]. The reduction of fatty acids intake generally has several benefits, such as a reduction in body weight, and cholesterol and triglyceride levels, and an improvement in the functioning of the myocardium [140,141]. Polyphenols are bioactive chemical compounds synthesized by plants that are mainly found in fruits and vegetables, where they are responsible for color, flavor, and many pharmacological activities. The very large family of polyphenols includes more than 8000 variants, classified according to their chemical structure into flavonoids (flavones, flavonols, isoflavones, neoflavonoids, chalcones, anthocyanidins, and proanthocyanidins) and non-flavonoids (phenolic acids, stilbenoids, and phenolic amides) [142]. Polyphenols are found mainly in fruits, vegetables, olive oil, nuts, seeds, roots, the leaves of different plants, herbs, whole wheat, red wine, coffee, and tea. Polyphenols do not arise from the primary metabolic reactions of plants, but from secondary metabolism [143]. This category of compound plays important metabolic roles in the human body, and interest in polyphenols has exponentially grown over the last two decades. The reasons for this growing attention are manifold: first, they are readily available and particularly safe for health, reduce the perishable nature of food and are consequently used to replace common synthetic food preservatives, and have beneficial properties in many aspects of human health. With regard to this last point, polyphenols demonstrate a wide range of biological activities, being also able to act synergistically; among these activities, they have antioxidant, anti-inflammatory, immunomodulating, antitumor, and protective benefits to the cardiovascular system [144,145]. To date, the recommended daily intake of polyphenols is in the range from 0.1 to 1.0 g [146]. This suggests that the long-term consumption of dietary polyphenols offers protection against the development of many diseases. The traditional Mediterranean diet is characterized by a high consumption of foods of vegetable origin, minimal quantities of red meat, and high quantities of olive oil and its derivatives. This particular choice is based on the need to reduce saturated and increase healthier unsaturated fats. The Mediterranean diet has been a winning strategy for maintaining health, and much experimental evidence showed a close correlation between the Mediterranean diet and a reduced incidence of developing cardiovascular diseases and cancer [147].

In general, the exact composition of plant derivatives is variable and depends on multiple factors including seasonality, the level of maturation of the product and the portion of plant used. Nevertheless, the composition of the product of interest can be identified with certainty by appropriate laboratory analysis. To date, the common techniques already known for the identification of volatile components of an extract, are based on the use of gas chromatography-mass spectrometry (GC-MS). The results obtained can be qualitatively better if metabolomic strategies are also associated [148]. Among natural compounds, bergamot (*Citrus bergamia*, Risso et Poiteu) has particular importance thanks to its countless pharmacological beneficial effects, including specific cardioprotective properties

The high levels of flavonoids contained in BPF formulation, refs. [61,62] showed important protective activities in the management of atherosclerosis, metabolic disorders, and cardiotoxicity, mainly due to its antioxidative, anti-inflammatory, and lipid-lowering effects [54,55,56,57]. In fact, clinical studies carried out on animal and cellular models showed that BPF has hypolipemic and antiatherogenic effects by interfering with the autophagic pathway and preventing pathogenic lipid accumulation [59,149,150]. In addition, BPF possesses powerful antioxidant effects, decreases lipid peroxidation biomarkers, and prevents ROS accumulation in different cell types [58,59,60]. BPF also improves the activity of endogenous antioxidant enzymes, including superoxide dismutase, glutathione peroxidase, and glutathione S transferase P1 [61]. Indeed, a recent double-blind study conducted on 60 patients with Type 2 diabetes mellitus and hyperlipemia identified interesting beneficial effects of BPF. In particular, the patients were divided into three groups: one received a placebo, the second received standard BPF, and the third received phytosomal formulation (BPF Phyto). The results obtained showed a significant reduction in fasting blood glucose, serum LDL cholesterol, and triglycerides, and an increase in HDL cholesterol in the group treated with BPF and BPF Phyto, thereby highlighting a hypolipemic and hypoglycemic effect of bergamot extract, both with the use of the standard formulation and BPF Phyto [62]. Although no differences in therapeutic response were observed between the BPF and BPF Phyto groups, a comparison of the pharmacokinetic profile of naringin (the main component of BPF) in patients treated with BPF Phyto showed an increase of at least 2.5-fold of its absorption, confirming a better profile of BPF Phyto compared to that of the BPF standard in human studies [62]. Another recent study highlighted the beneficial effects of citrus-derived polyphenols on postprandial blood glucose and insulin in healthy individuals [46]. A group of volunteers were given breakfast based on a brioche enriched with wheat bran and bergamot fiber, comparing it to the consumption of a canonical brioche. The results obtained showed that the association between wheat bran and bergamot fiber significantly affected glucose metabolism, exerting insulin-like effects [46]. The hypothesis of these results, if confirmed in a larger study, may suggest a strategy to control the glycometabolic status in patients with Type 2 diabetes [150]. Analyzing the pathophysiological mechanisms of diabetic cardiomyopathy, especially the onset of oxidative stress, mitochondrial and SR dysfunctions were evidenced [151,152]. Under these conditions, protective mechanisms that include the overexpression of endogenous antioxidant enzymes, UPR, and autophagic responses are activated with the aim of antagonizing the apoptotic cell death of myocardial cells [153]. Since BPF counteracts oxidative stress in in vitro and in vivo cardiotoxicity models [49,50,53], in addition to reverting mitochondrial dysfunction [94,148,153], it is likely to assume its protective role even in the early stages of diabetic cardiomyopathy, in cardiac dysfunction, and in countering the development of heart failure.

It should be noted that other compounds belonging to the genus citrus show interesting pharmacological effects on cardiovascular protection. Citrus flavonoids that should be mentioned are nobiletin, hesperidin, hesperetin, rutin, tangeretin, eriodictyol and others [154,155].

### 2.1. Beneficial Properties of Bergamot Polyphenols on the Sarcoplasmic Reticulum in Diabetic Cardiomyopathy

The main compounds of citrus fruits, contained in particularly high percentages in the BPF formulation, are naringin, neoeriocitrin, neohesperidin, and glycosylated polyphenols, such as bruteridin and melitidin [156,157]. Among these, naringin is the component present in greater quantity. Naringin has numerous pharmacological activities, including antilipidemic [158], antiatherogenic, superoxide scavenging, antioxidant, and anti-inflammatory activities [159]. Naringin is a flavanone glycoside and has a chemical formula of C_27_H_32_O_14_. It is formed from the flavanone naringenin and the disaccharide neohesperidose; naringin can be found in a diversity of vegetables (tomatoes, beans, Greek oregano, cocoa), beverages (red wine, tea, coffee, water mint), fruits (grapefruit, sour orange, bergamot) and other citrus fruits (*Citrus aurantium* L. and *Citrus medica* L.). In the case of citrus fruits, naringin is responsible for the bitter taste of the juices of these fruits [160]. The naringin content of fruit depends on a number of factors including the degree of ripeness at harvest, the season, the part of fruit used, the washing and drying time of the fruit. For this reason, it is difficult to affirm the exact content of naringin in every fruit without incurring errors or inaccuracies. What can be said without any doubt is that a ripe citrus contains a greater amount of naringin than the same fruit that has not reached a similar level of ripeness [161]. Naringin is isolated by three sequential steps: extraction, separation and purification. In general, the main components are obtained from citrus fruits based on their UV and mass spectra. A method based on microwave assisted extraction (MAE) and high-speed counter-current chromatography (HSCCC) allows the extraction of about 200 mg of naringin, at a purity of 97% to 99.5%, from 1.5 g of crude citrus extract: naringin thus represents 13–15% of the citrus fruits concerned [162,163]. In general, it is possible to affirm that, per kg of bergamot fruit, we have 1 g of naringin, while in one Kg of BPF we have 140 g of narinigin [51,55]. Although these data are fairly approximate, due to the above-described naringin content variation factors, the percentage of naringin in BPF is confirmed at about 14%. Since the naringin LD_50_ is about 2000 mg/kg and and because of its inhibitory effect on liver enzymes (cytochrome P450 enzymes), its high consumption can increase the concentration of drugs that are metabolized in the liver altering the pharmacokinetics and leading to toxicity [164].

The effect of naringin was tested in vivo on streptozotocin-induced diabetic rats, which eventually developed diabetic cardiomyopathy. Indeed, the induction of diabetes caused changes in myocardial tissue, increased the content of malonildialdehyde (MDA), decreased superoxide dismutase (SOD) enzyme activity, and increased protein expression of GRP78 and CHOP, related to SR stress, and caspase 12, linked to apoptotic death [165]. In this experimental model, naringin treatment alleviated damage to the myocardial structure, decreased the content of MDA, and significantly increased SOD activities. In addition, the protein expressions of GRP78 and CHOP were decreased, demonstrating a protective action of naringin on SR stress of on mitochondrial oxidative stress induced by diabetes [166]. In parallel, an in vitro study was conducted on vascular endothelial cells in which a condition of stress that was induced by serum starvation and naringin was tested under the same experimental conditions at different concentrations and for different times [167]. In this case, protection against impaired SR was highlighted. These results showed that the protective action of naringin on SR occurs regardless of the type of damage that generated it [168]. Many cellular alterations present in the myocardium of patients suffering from diabetic cardiomyopathy, including cardiac-cell apoptosis, oxidative stress, mitochondrial damage, SR damage, proinflammatory cytokine production, myocardial hypertrophy, myocardial remodeling, and cardiac fibrosis are related to the activation of the nuclear factor kappa B (NF-κB) pathway both in vitro and in vivo: in fact, transcription factor NF-κB controls most of the molecular processes within cardiomyocytes, and its dysregulation is involved in many cardiovascular diseases [169]. Normally, NF-kB remains in the cell cytoplasm, bound to the IkB factor that keeps it inactive. The activation of the NF-κB pathway involves its detachment from IkB and translocation into the nucleus, where a specific subunit binds to some specific genes regulating their expression [170]. The same pathophysiologic process occurs in cardiomyocyte injuries induced by hyperglycemia. The cardioprotection of naringin in an experimental hyperglycemia model was demonstrated [171]. In particular, the following mechanisms are activated: (1) nargirin activates glutathione (GSH), ensuring a new antioxidant defense mechanism; (2) naringin prevents the progression of hyperglycemia by increasing hepatic glycolysis and lowering hepatic gluconeogenesis; (3) nargirin shows antidiabetic effects; (4) naringin inhibits the hyperglycemia-induced NF-κB pathway [156]. The involvement of SR stress in diabetic cardiomyopathy was also demonstrated by the important results obtained in an in vitro study on H9c2 embryonic rat cardiomyoblast maintained in culture and exposed to high glucose concentrations [172]. In this study, when the cells were exposed to exenatide, a drug used in the treatment of Type 2 diabetes mellitus, the inhibition of the NF-κB signaling pathway was observed, reducing SR stress induced by hyperglycemia [162]. Since naringin, in addition to exenatide, can inhibit the NF-κB signaling pathway and thus reverse SR stress, this natural constituent of BPF could be a valid substitute and/or support for the treatment of diabetic cardiomyopathy.

#### Metabolism of Polyphenols of Bergamot

After ingestion, flavonoids undergo extensive metabolization and absorption. These phases occur in both the small and large intestines and a substantial assumed fraction reaches the colon where flavonoids are exposed to the microbiota. In particular, the microbiome catabolizes unabsorbed flavonoids, by hydrolysis and fermentation, into smaller molecules that can become bioavailable. Flavonoids initially undergo phase I metabolic reactions and the resulting metabolites are transported to the liver. In this organ they undergo a further phase I metabolism followed by a series of phase II metabolic reactions that transform the compounds into more polar structures, called glucoronides. These molecules can be excreted through the kidneys in the urine or through the bile or transported back into the intestinal lumen [173,174]. Recent literature studies have indicated in endothelial cells that, following the intake of bergamot juice, 12 metabolites were identified in plasma and urine samples of the volunteers: 5 metabolites were esperetin conjugates, 4 naringenin conjugates, and 3 eriodictyol derivatives. It is interesting to note that these metabolites, to varying degrees, have been found to reduce lipotoxic damage by decreasing the gene expression of certain inflammatory cytokines (IL-1β, TNF-α and IL-8); in addition none of the tested metabolites induced cytotoxic effects [175,176]. In order to evaluate cellular and molecular mechanisms of action of flavanoids metabolites on endothelial cells, further studies have been carried out. A well known paper reported that naringenin and hesperetin metabolites (hesperetin-3′-sulphate, hesperetin-3′-glucuronide and naringenin-4′- glucuronide, at concentration of 2 mM, were able to modulated the expression of genes involved in atherogenesis, including those involved in inflammation, cytoskeletal organisation and cell adhesion, providing a vasculoprotective effects of flavanones [177]. To confirm these data, another important scientific work has evidenced, in human aortic endothelial cell (HAEC), that glucuronides and sulfates metabolites of flavonoids (hesperetin 3′O-glucuronide, hesperetin 7′O-glucuronide, hesperetin 3′O-sulfate, hesperetin 7′O-sulfate and hesperetin) at physiological concentrations where they are found after metabolism (1–10 um) are able to attenuate cell migration, decrease the levels of thrombogenic plasminogen activator inhibitor-1 (PAI-1) and reduce pro-inflammatory stimulus. Although further studies are needed, it can be concluded that, following consumption of bergamot juice, circulating phase-II flavonoids metabolites can contribute to cardioprotective effects [178].

## 3. Discussion and Conclusions

In summary, myocardial dysfunction is mainly caused by a condition of frailty of the cardiomyocytes that, under stress, can collide with the physiological loss of the main cellular organelles, such as mitochondria and SR [179,180,181,182,183]. In the early stage of diabetic cardiomyopathy, there are collective metabolic disorders that promote cardiac remodeling and fibrotic diastolic dysfunction. In the later stages, changes in cardiac structure are more pronounced, and the functional dysfunction of the myocardium, reduction in the ejection fraction, and cardiomyocyte necrosis occur [184,185]. There are many mechanisms that contribute to reduced heart performance in diabetic cardiomyopathy. Among these, a key role is played by hyperglycemia with the increase in fatty acids and cytokines. In this condition, exposure to increased lipid levels leads to cardiac lipotoxicity [156,186]. The formation of non-enzymatic advanced glycation end products also increases the harmful effects. In particular, advanced glycation products activate the NADPH oxidase, triggering the formation of peroxide and the accumulation of ROS, which are responsible for the DNA damage of cardiomyocytes [187]. Mitochondrial oxidative stress and Ca^2+^ alteration, caused by SR dysfunction, significantly contribute to diabetic cardiomyopathy. The therapy of diabetic cardiomyopathy includes the simultaneous use of different drugs, able to improve multiple involved aspects, although antihyperglycemic drugs remain essential in the management of the disease, effectively reducing complications [188]. An increase in fruit and vegetable intake can affect carbohydrate and lipid metabolism, and regulate genes related to the pathophysiology of diabetic cardiomyopathy, reducing myocardial dysfunction and cardiomyocyte death [189]. Furthermore, several epidemiological experiments showed that natural compounds reduce the risk of suffering cardiovascular diseases [190,191]. The literature suggests that the bergamot fruit (*Citrus bergamia* Risso et Poiteau), 80% of which is produced in Calabria, southern Italy, is composed of a high percentage of polyphenols [192] and plays an important role in several areas of interest, including cardiovascular health, diabetes, inflammation, the nervous system, bone metabolism, and skin [193]. Naringin, the main component of BPF, is able to reverse SR stress, induced in diabetic cardiomyopathy. Since BPF has anti-inflammatory, antioxidant [94], hypoglycemic, and hypolipemic effects [46,53,58,59,150,152,153], in this review, we further examined the beneficial role of BPF on the pathophysiological mechanisms of diabetic cardiomyopathy. If the protective effects of BPF are further confirmed in clinical trials with a large number of enrolled patients, a greater contribution could be made in the management of diabetic cardiomyopathy through nutraceutical supplementation and the use of optimal micronutrients.

## References

[1] Kyu H.H., Abate D., Abate K.H., Abay S.M., Abbafati C., Abbasi N., Abbastabar H., Abd-Allah F., Abdela J., Abdelalim A. (2018). Global, regional, and national disability-adjusted life-years (DALYs) for 359 diseases and injuries and healthy life expectancy (HALE) for 195 countries and territories, 1990–2017: A systematic analysis for the global burden of disease study. Lancet.

[2] Haidar M.N., Islam M.B., Chowdhury U.N., Rahman M.R., Huq F., Quinn J.M.W., Moni M.A. (2020). Network-based computational approach to identify genetic links between cardiomyopathy and its risk factors. IET Syst. Biol..

[3] Pluijmert N.J., den Haan M.C., van Zuylen V.L., Steendijk P., de Boer H.C., van Zonneveld A.J., Fibbe W.E., Schalij M.J., Quax P.H.A., Atsma D.E. (2019). Hypercholesterolemia affects cardiac function, infarct size and inflammation in APOE*3-Leiden mice following myocardial ischemia-reperfusion injury. PLoS ONE.

[4] Liu S., Lin X., Shi X., Fang L., Huo L., Shang F., Knuuti J., Han C., Wu X., Guo R. (2020). Myocardial tissue and metabolism characterization in men with alcohol consumption by cardiovascular magnetic resonance and 11C-acetate PET/CT. J. Cardiovasc. Magn. Reson..

[5] Abshire M., Xu J., Baptiste D.-L., Almansa J.R., Xu J., Cummings A., Andrews M.J., Himmelfarb C.D. (2015). Nutritional Interventions in Heart Failure: A Systematic Review of the Literature. J. Card. Fail..

[6] Tsioufis C., Georgiopoulos G., Oikonomou D., Thomopoulos C., Katsiki N., Kasiakogias A., Chrysochoou C., Konstantinidis D., Kalos T., Tousoulis D. (2017). Hypertension and Heart Failure with Preserved Ejection Fraction: Connecting the Dots. Curr. Vasc. Pharmacol..

[7] Piccand E., Vollenweider P., Guessous I., Marques-Vidal P. (2018). Association between dietary intake and inflammatory markers: Results from the CoLaus study. Public Health Nutr..

[8] Badimon L., Chagas P., Chiva-Blanch G. (2019). Diet and Cardiovascular Disease: Effects of Foods and Nutrients in Classical and Emerging Cardiovascular Risk Factors. Curr. Med. Chem..

[9] Gliozzi M., Musolino V., Bosco F., Scicchitano M., Scarano F., Nucera S., Zito M.C., Ruga S., Carresi C., Macrì R. (2021). Cholesterol homeostasis: Researching a dialogue between the brain and peripheral tissues. Pharmacol. Res..

[10] Giglio R.V., Patti A.M., Cicero A.F.G., Lippi G., Rizzo M., Toth P.P., Banach M. (2018). Polyphenols: Potential Use in the Prevention and Treatment of Cardiovascular Diseases. Curr. Pharm. Des..

[11] Brieler J., Breeden M.A., Tucker J. (2017). Cardiomyopathy: An Overview. Am. Fam. Physician.

[12] Mazzarotto F., Olivotto I., Boschi B., Girolami F., Poggesi C., Barton P.J.R., Walsh R. (2020). Contemporary Insights Into the Genetics of Hypertrophic Cardiomyopathy: Toward a New Era in Clinical Testing?. J. Am. Heart Assoc..

[13] Ciutac A.M., Dawson D. (2021). The role of inflammation in stress cardiomyopathy. Trends Cardiovasc. Med..

[14] Antunes M.O., Scudeler T.L. (2020). Hypertrophic cardiomyopathy. Int. J. Cardiol. Heart Vasc..

[15] Casas R., Castro-Barquero S., Estruch R., Sacanella E. (2018). Nutrition and Cardiovascular Health. Int. J. Mol. Sci..

[16] De Rosa S., Arcidiacono B., Chiefari E., Brunetti A., Indolfi C., Foti D.P. (2018). Type 2 Diabetes Mellitus and Cardiovascular Disease: Genetic and Epigenetic Links. Front Endocrinol..

[17] Adeghate E., Singh J. (2014). Structural changes in the myocardium during diabetes-induced cardiomyopathy. Heart Fail Rev..

[18] Fang Z.Y., Prins J.B., Marwick T.H. (2004). Diabetic cardiomyopathy: Evidence, mechanisms, and therapeutic implications. Endocr. Rev..

[19] Mytas D.Z., Stougiannos P.N., Zairis M.N., Foussas S.G., Pyrgakis V.N., Kyriazis I.A. (2009). Diabetic myocardial disease: Pathophysiology, early diagnosis and therapeutic options. J. Diabetes Complicat..

[20] Wang J., Song Y., Wang Q., Kralik P.M., Epstein P.N. (2006). Causes and characteristics of diabetic cardiomyopathy. Rev. Diabet. Stud..

[21] Lazo M., Halushka M., Shen L., Maruthur N., Rebholz C.M., Rawlings A., Hoogeveen R., Brinkley T.E., Ballantyne C.M., Astor B.C. (2015). Soluble receptor for advanced glycation end products and the risk for incident heart failure: The Atherosclerosis Risk in Communities Study. Am. Heart J..

[22] Jia G., Habibi J., DeMarco V.G., Martinez-Lemus L.A., Ma L., Whaley-Connell A.T., Aroor A.R., Domeier T.L., Zhu Y., Meininger G.A. (2015). Endothelial mineralocorticoid receptor deletion prevents diet-induced cardiac diastolic dysfunction in females. Hypertension.

[23] Ljubkovic M., Gressette M., Bulat C., Cavar M., Bakovic D., Fabijanic D., Grkovic I., Lemaire C., Marinovic J. (2019). Disturbed Fatty Acid Oxidation, Endoplasmic Reticulum Stress, and Apoptosis in Left Ventricle of Patients With Type 2 Diabetes. Diabetes.

[24] Berceanu M., Mirea O., Târtea G.C., Donoiu I., Militaru C., Istrătoaie OSăftoiu A. (2019). The Significance of Right Ventricle in Young Subjects with Diabetes Mellitus Type 1. An echocardiographyic study. Curr. Health Sci. J..

[25] Kolwicz S.C., Purohit S., Tian R. (2013). Cardiac metabolism and its interactions with contraction, growth, and survival of cardiomyocytes. Circ. Res..

[26] Eisner V., Csordás G., Hajnóczky G. (2013). Interactions between sarco-endoplasmic reticulum and mitochondria in cardiac and skeletal muscle-pivotal roles in Ca^2+^ and reactive oxygen species signaling. J. Cell Sci..

[27] Bravo-Sagua R., Parra V., Muñoz-Cordova F., Sanchez-Aguilera P., Garrido V., Contreras-Ferrat A., Chiong M., Lavandero S. (2020). Sarcoplasmic reticulum and calcium signaling in muscle cells: Homeostasis and disease. Int. Rev. Cell Mol. Biol..

[28] Lacroix S., Cantin J., Nigam A. (2017). Contemporary issues regarding nutrition in cardiovascular rehabilitation. Ann. Phys. Rehabil. Med..

[29] Cvetinovic N., Loncar G., Isakovic A.M., von Haehling S., Doehner W., Lainscak M., Farkas J. (2019). Micronutrient Depletion in Heart Failure: Common, Clinically Relevant and Treatable. Int. J. Mol. Sci..

[30] Lopresti A.L. (2020). Association between Micronutrients and Heart Rate Variability: A Review of Human Studies. Adv. Nutr..

[31] Oppedisano F., Macrì R., Gliozzi M., Musolino V., Carresi C., Maiuolo J., Bosco F., Nucera S., Zito M.C., Guarnieri L. (2020). The Anti-Inflammatory and Antioxidant Properties of n-3 PUFAs: Their Role in Cardiovascular Protection. Biomedicines.

[32] Gliozzi M., Scarano F., Musolino V., Carresi C., Scarcella A., Nucera S., Scicchitano M., Ruga S., Bosco F., Maiuolo J. (2021). Paradoxical effect of fat diet in matrix metalloproteinases induced mitochondrial dysfunction in diabetic cardiomyopathy. J. Cardiovasc. Med..

[33] Chen D., Li X., Zhang L., Zhu M., Gao L. (2018). A high-fat diet impairs mitochondrial biogenesis, mitochondrial dynamics, and the respiratory chain complex in rat myocardial tissues. J. Cell Biochem..

[34] Bianchi V.E. (2020). Nutrition in chronic heart failure patients: A systematic review. Heart Fail Rev..

[35] Siri-Tarino P.W., Krauss R.M. (2016). Diet, lipids, and cardiovascular disease. Curr. Opin Lipidol..

[36] Bach-Faig A., Berry E.M., Lairon D., Reguant J., Trichopoulou A., Dernini S., Medina F.X., Battino M., Belahsen R., Miranda G. (2011). Mediterranean Diet Foundation Expert Group. Mediterranean diet pyramid today. Science and cultural updates. Public Health Nutr..

[37] Valls-Pedret C., Sala-Vila A., Serra-Mir M., Corella D., de la Torre R., Martínez-González M.Á., Ros E. (2015). Mediterranean Diet and Age-Related Cognitive Decline: A Randomized Clinical Trial. JAMA Intern. Med..

[38] Estruch R., Ros E., Salas-Salvadó J., Covas M.I., Corella D., Arós F., Martínez-González M.A. (2013). PREDIMED Study Investigators. Primary prevention of cardiovascular disease with a Mediterranean diet. N. Engl. J. Med..

[39] Estruch R., Ros E., Salas-Salvadó J., Covas M.I., Corella D., Arós F., Gómez-Gracia E., Ruiz-Guntieerez V., Fiol M. (2018). Primary Prevention of Cardiovascular Disease with a Mediterranean Diet Supplemented with Extra-Virgin Olive Oil or Nuts. PREDIMED Study Investigators. N. Engl. J. Med..

[40] Salerno R., Casale F., Calandruccio C., Procopio A. (2016). Characterization of flavonoids in Citrus bergamia (Bergamot) polyphenolic fraction by liquid chromatography-high resolution mass spectrometry (LC/HRMS). PharmaNutrition.

[41] Corasaniti M.T., Maiuolo J., Maida S., Fratto V., Navarra M., Russo R., Amantea D., A Morrone L., Bagetta G. (2007). Cell signaling pathways in the mechanisms of neuroprotection afforded by bergamot essential oil against NMDA-induced cell death in vitro. Br. J. Pharmacol..

[42] Bagetta G., Morrone L.A., Rombolà L., Amantea D., Russo R., Berliocchi L., Sakurada S., Sakurada T., Rotiroti D., Corasaniti M.T. (2010). Neuropharmacology of the essential oil of bergamot. Fitoterapia.

[43] Navarra M., Ferlazzo N., Cirmi S., Trapasso E., Bramanti P., Lombardo G.E., Minciullo P.L., Calapai G., Gangemi S. (2015). Effects of bergamot essential oil and its extractive fractions on SH-SY5Y human neuroblastoma cell growth. J. Pharm. Pharmacol..

[44] Pujia A., Russo C., Maurotti S., Pujia R., Mollace V., Romeo S., Montalcini T. (2018). Bergamot Polyphenol Fraction Exerts Effects on Bone Biology by Activating ERK 1/2 and Wnt/β-Catenin Pathway and Regulating Bone Biomarkers in Bone Cell Cultures. Nutrients.

[45] Nisticò S., Ehrlich J., Gliozzi M., Maiuolo J., Del Duca E., Muscoli C., Mollace V. (2015). Telomere and telomerase modulation by bergamot polyphenolic fraction in experimental photoageing in human keratinocytes. J. Biol. Regul. Homeost. Agents.

[46] Mare R., Mazza E., Ferro Y., Gliozzi M., Nucera S., Paone S., Aversa I., Pujia R., Marafioti G., Musolino V. (2020). A new breakfast brioche containing bergamot fiber prevents insulin and glucose increase in healthy volunteers: A pilot study. Minerva Endocrinol..

[47] Cappello A.R., Dolce V., Iacopetta D., Martello M., Fiorillo M., Curcio R., Muto L., Dhanyalayam D. (2016). Bergamot (Citrus bergamia Risso) Flavonoids and Their Potential Benefits in Human Hyperlipidemia and Atherosclerosis: An Overview. Mini Rev. Med. Chem..

[48] Scarano F., Gliozzi M., Zito M., Guarnieri L., Carresi C., Macrì R., Nucera S., Scicchitano M., Bosco F., Ruga S. (2021). Potential of Nutraceutical Supplementation in the Modulation of White and Brown Fat Tissues in Obesity-Associated Disorders: Role of Inflammatory Signalling. Int. J. Mol. Sci..

[49] Carresi C., Scicchitano M., Scarano F., Macrì R., Bosco F., Nucera S., Mollace V. (2021). The Potential Properties of Natural Compounds in Cardiac Stem Cell Activation: Their Role in Myocardial Regeneration. Nutrients.

[50] Perna S., Spadaccini D., Botteri L., Girometta C., Riva A., Allegrini P., Petrangolini G., Infantino V., Rondanelli M. (2019). Efficacy of bergamot: From anti-inflammatory and anti-oxidative mechanisms to clinical applications as preventive agent for cardiovascular morbidity, skin diseases, and mood alterations. Food Sci. Nutr..

[51] Olas B. (2020). Honey and Its Phenolic Compounds as an Effective Natural Medicine for Cardiovascular Diseases in Humans?. Nutrients.

[52] Musolino V., Gliozzi M., Nucera S., Carresi C., Maiuolo J., Mollace R., Paone S., Bosco F., Scarano F., Scicchitano M. (2019). The effect of bergamot polyphenolic fraction on lipid transfer protein system and vascular oxidative stress in a rat model of hyperlipemia. Lipids Health Dis..

[53] Vincenzo M., V M., Mollace R., Gliozzi M., Tavernese A., Musolino V., CarresiDaly C., ScicchitanoFoote M., Palma E., Nucera S. (2018). Bergamot Polyphenolic Fraction supplementation improves metabolic balance, endothelial function and maximal oxygen uptake in athletes. J. Sports Med. Ther..

[54] Carresi C., Gliozzi M., Musolino V., Scicchitano M., Scarano F., Bosco F., Nucera S., Maiuolo J., Macrì R., Ruga S. (2020). The Effect of Natural Antioxidants in the Development of Metabolic Syndrome: Focus on Bergamot Polyphenolic Fraction. Nutrients.

[55] Musolino V., Gliozzi M., Scarano F., Bosco F., Scicchitano M., Nucera S., Carresi C., Ruga S., Zito M.C., Maiuolo J. (2020). Bergamot Polyphenols Improve Dyslipidemia and Pathophysiological Features in a Mouse Model of Non-Alcoholic Fatty Liver Disease. Sci. Rep..

[56] Carresi C., Musolino V., Gliozzi M., Maiuolo J., Mollace R., Nucera S., Maretta A., Sergi D., Muscoli S., Gratteri S. (2018). Anti-oxidant effect of bergamot polyphenolic fraction counteracts doxorubicin-induced cardiomyopathy: Role of autophagy and c-kit^pos^CD45^neg^CD31^neg^ cardiac stem cell activation. J. Mol. Cell Cardiol..

[57] La Russa D., Giordano F., Marrone A., Parafati M., Janda E., Pellegrino D. (2019). Oxidative Imbalance and Kidney Damage in Cafeteria Diet-Induced Rat Model of Metabolic Syndrome: Effect of Bergamot Polyphenolic Fraction. Antioxidants.

[58] Gliozzi M., Walker R., Muscoli S., Vitale C., Gratteri S., Carresi C., Mollace V. (2013). Bergamot polyphenolic fraction enhances rosuvastatin-induced effect on LDL-cholesterol, LOX-1 expression and protein kinase B phosphorylation in patients with hyperlipidemia. Int. J. Cardiol..

[59] Gliozzi M., Carresi C., Musolino V., Palma E., Muscoli C., Vitale C., Gratteri S., Muscianisi G., Janda E., Muscoli S. (2014). The effect of bergamot-derived polyphenolic fraction on LDL small dense particles and non-alcoholic fatty liver disease in patients with metabolic syndrome. Adv. Biol. Chem..

[60] Mollace V., Sacco I., Janda E., Malara C., Ventrice D., Colica C., Romeo F. (2011). Hypolipemic and hypoglycaemic activity of bergamot polyphenols: From animal models to human studies. Fitoterapia.

[61] Mollace V., Scicchitano M., Paone S., Casale F., Calandruccio C., Gliozzi M., Musolino V., Carresi C., Maiuolo J., Nucera S. (2019). Hypoglycemic and Hypolipemic Effects of a New Lecithin Formulation of Bergamot Polyphenolic Fraction: A Double Blind, Randomized, Placebo-Controlled Study. Endocr. Metab. Immune Disord. Drug Targets.

[62] Dadson K., Hauck L., Billia F. (2017). Molecular mechanisms in cardiomyopathy. Clin. Sci..

[63] Duncker D., König T., Hohmann S., Veltmann C. (2015). Primary and secondary prophylactic ICD therapy in congenital electrical and structural cardiomyopathies. Herzschrittmacherther Elektrophysiol..

[64] Tomczyk M.M., Dolinsky V.W. (2020). The Cardiac Lipidome in Models of Cardiovascular Disease. Metabolites.

[65] Sun J., Han S., Hu J., Jiang C., Wang Q., Zheng L., Zhou Z., Qi M., Writing Group For Practice Guidelines For Diagnosis And Treatment Of Genetic Diseases Medical Genetics Branch Of Chinese Medical Association (2020). Clinical practice guidelines for hereditary cardiomyopathy. Zhonghua Yi Xue Yi Chuan Xue Za Zhi.

[66] Paulussen K.J.M., McKenna C.F., Beals J.W., Wilund K.R., Salvador A.F., Burd N.A. (2021). Anabolic Resistance of Muscle Protein Turnover Comes in Various Shapes and Sizes. Front. Nutr..

[67] Glatz J.F.C., Luiken J.F.P. (2017). From Fat to FAT (CD36/SR-B2): Understanding the Regulation of Cellular Fatty Acid Uptake. Biochimie.

[68] Zhang X., Liu C., Liu C., Wang Y., Zhang W., Xing Y. (2019). Trimetazidine and l-carnitine prevent heart aging and cardiac metabolic impairment in rats via regulating cardiac metabolic substrates. Exp. Gerontol..

[69] Lopaschuk G.D., Ussher J.R., Folmes C.D.L., Jaswal J.S., Stanley W.C. (2010). Myocardial fatty acid metabolism in health and disease. Physiol. Rev..

[70] Yang X., Rodriguez M., Leonard A., Sun L., Fischer K.A., Wang Y., Murry C.E. (2019). Fatty Acids Enhance the Maturation of Cardiomyocytes Derived From Human Pluripotent Stem Cells. Stem Cell Rep..

[71] Gandoy-Fieiras N., Gonzalez-Juanatey J.R., Eiras S. (2020). Myocardium Metabolism in Physiological and Pathophysiological States: Implications of Epicardial Adipose Tissue and Potential Therapeutic Targets. Int. J. Mol. Sci..

[72] Yang Q., Li Y. (2007). Roles of PPARs on regulating myocardial energy and lipid homeostasis. J. Mol. Med..

[73] Bowen T.S., Rolim N.P.L., Fischer T., Baekkerud F.H., Medeiros A., Werner S., Brønstad E., Rognmo O., Mangner N., Linke A. (2015). Heart failure with preserved ejection fraction induces molecular, mitochondrial, histological, and functional alterations in rat respiratory and limb skeletal muscle. Eur. J. Heart Fail..

[74] Roul D., Recchia F.A. (2015). Metabolic Alterations Induce Oxidative Stress in Diabetic and Failing Hearts: Different Pathways, Same Outcome. Antioxid. Redox Signal..

[75] Gupte S.A., Levine R.J., Gupte R.S., Young M.E., Lionetti V., Labinskyy V., Floyd B.C., Ojaimi C., Bellomo M., Wolin M.S. (2006). Glucose-6-phosphate dehydrogenase-derived NADPH fuels superoxide production in the failing heart. J. Mol. Cell Cardiol..

[76] Vimercati C., Qanud K., Mitacchione G., Sosnowska D., Ungvari Z., Sarnari R., Mania D., Patel N., Hintze T.H., Gupte S.A. (2014). Beneficial effects of acute inhibition of the oxidative pentose phosphate pathway in the failing heart. Am. J. Physiol. Heart Circ. Physiol..

[77] Scolletta S., Biagioli B. (2010). Energetic myocardial metabolism and oxidative stress: Let’s make them our friends in the fight against heart failure. Biomed. Pharmacother..

[78] Zheng J., Cheng J., Zheng S., Zhang L., Guo X., Zhang J., Xiao X. (2018). Physical Exercise and Its Protective Effects on Diabetic Cardiomyopathy: What Is the Evidence?. Front Endocrinol..

[79] Pregnolato M., Damiani G., Pereira A. (2017). Patterns of Calcium Signaling: A Link between Chronic Emotions and Cancer. J. Integr. Neurosci..

[80] Guaricci A., Bulzis G., Pontone G., Scicchitano P., Carbonara R., Rabbat M., De Santis D., Ciccone M.M. (2018). Current Interpretation of Myocardial Stunning. Trends Cardiovasc. Med..

[81] Dewenter M., von der Lieth A., Katus H.A., Backs J. (2017). Calcium Signaling and Transcriptional Regulation in Cardiomyocytes. Circ. Res..

[82] Romero-Garcia S., Prado-Garcia H. (2019). Mitochondrial Calcium: Transport and Modulation of Cellular Processes in Homeostasis and Cancer. Int. J. Oncol..

[83] Mohamed B.A., Hartmann N., Tirilomis P., Sekeres K., Li W., Neef S., Toischer K. (2018). Sarcoplasmic Reticulum Calcium Leak Contributes to Arrhythmia but Not to Heart Failure Progression. Sci. Transl. Med..

[84] Chen Y., Hua Y., Li X., Arslan I.M., Zhang W., Meng G. (2020). Distinct Types of Cell Death and the Implication in Diabetic Cardiomyopathy. Front. Pharmacol..

[85] Kumar A.A., Kelly D.P., Chirinos J.A. (2019). Mitochondrial Dysfunction in Heart Failure With Preserved Ejection Fraction. Circulation..

[86] Zhao D., Yang J., Yang L. (2017). Insights for Oxidative Stress and mTOR Signaling in Myocardial Ischemia/Reperfusion Injury under Diabetes. Oxidative Med. Cell Longev..

[87] Christen F., Desrosiers V., Dupont-Cyr B.A., Vandenberg G.W., Le François N.R., Tardif J.C., Dufresne F., Lamarre S.G., Blier P.U. (2018). Thermal tolerance and thermal sensitivity of heart mitochondria: Mitochondrial integrity and ROS production. Free Radic. Biol. Med..

[88] Tahrir F.G., Langford D., Amini S., Mohseni A.T., Khalili K. (2019). Mitochondrial quality control in cardiac cells: Mechanisms and role in cardiac cell injury and disease. J. Cell Physiol..

[89] Picca A., Mankowski R.T., Burman J.L., Donisi L., Kim J.S., Marzetti E., Leeuwenburgh C. (2018). Mitochondrial quality control mechanisms as molecular targets in cardiac ageing. Nat. Rev. Cardiol..

[90] Fan H., He Z., Huang H., Zhuang H., Liu H., Liu X., Yang S., He P., Yang H., Feng D. (2020). Mitochondrial Quality Control in Cardiomyocytes: A Critical Role in the Progression of Cardiovascular Diseases. Front. Physiol..

[91] Pickles S., Vigié P., Youle R.J. (2018). Mitophagy and quality control mechanisms in mitochondrial maintenance. Curr. Biol..

[92] Westermann B. (2010). Mitochondrial fusion and fission in cell life and death. Nature reviews Mol. Cell Biol..

[93] Zhu X., Shen W., Yao K., Wang H., Liu B., Li T., Ju Z. (2019). Fine-Tuning of PGC1α Expression Regulates Cardiac Function and Longevity. Circ. Res..

[94] Warren J., Tracy C.M., Miller M.R., Makaju A., Szulik M.W., Oka S., Yuzyuk T.N., Cox J.E., Kumar A., Lozier B.K. (2018). Histone Methyltransferase Smyd1 Regulates Mitochondrial Energetics in the Heart. Proc. Natl. Acad. Sci. USA.

[95] Kasai S., Shimizu S., Tatara Y., Mimura J., Itoh K. (2020). Regulation of Nrf2 by Mitochondrial Reactive Oxygen Species in Physiology and Pathology. Biomolecules.

[96] Maiuolo J., Maretta A., Gliozzi M., Musolino V., Carresi C., Bosco F., Mollace R., Scarano F., Palma E., Scicchitano M. (2018). Ethanol-induced cardiomyocyte toxicity implicit autophagy and NFkB transcription factor. Pharmacol. Res..

[97] Chimienti G., Picca A., Sirago G., Fracasso F., Calvani R., Bernabei R., Lezza A.M.S. (2018). Increased TFAM Binding to mtDNA Damage Hot Spots Is Associated With mtDNA Loss in Aged Rat Heart. Free Radic. Biol. Med..

[98] Luo G., Jian Z., Zhu Y., Zhu Y., Chen B., Ma R., Tang R., Xiao Y. (2019). Sirt1 Promotes Autophagy and Inhibits Apoptosis to Protect Cardiomyocytes From Hypoxic Stress. J. Mol. Med..

[99] Sharma K. (2015). Mitochondrial hormesis and diabetic complications. Diabetes.

[100] Lee T.W., Bai K.J., Lee T.I., Chao T.F., Kao Y.H., Chen Y.J. (2017). PPARs modulate cardiac metabolism and mitochondrial function in diabetes. J. Biomed. Sci..

[101] Gao P., Yan Z., Zhu Z. (2020). Mitochondria-Associated Endoplasmic Reticulum Membranes in Cardiovascular Diseases. Front. Cell Dev. Biol..

[102] Ríos E. (2017). Perspectives on “Control of Ca release from within the cardiac sarcoplasmic reticulum”. J. Gen. Physiol..

[103] Yamaguchi N. (2020). Molecular Insights Into Calcium Dependent Regulation of Ryanodine Receptor Calcium Release Channels. Adv. Exp. Med. Biol..

[104] Laver D.R. (2018). Regulation of the RyR Channel Gating by Ca^2+^and Mg^2+^. Biophys. Rev..

[105] Meissner G.J. (2017). The structural basis of ryanodine receptor ion channel function. Gen. Physiol..

[106] Gong D., Chi X., Wei J., Zhou G., Huang G., Zhang L., Wang R., Lei J., Chen S.W., Yan N. (2019). Modulation of cardiac ryanodine receptor 2 by calmodulin. Nature.

[107] Arrieta A., Blackwood E.A., Stauffer W.T., Glembotski C.C. (2020). Integrating ER and Mitochondrial Proteostasis in the Healthy and Diseased Heart. Front. Cardiovasc. Med..

[108] Arrieta A., Blackwood E.A., Glembotski C.C. (2018). ER protein quality control and the unfolded protein response in the heart. Curr. Top. Microbiol. Immunol..

[109] Valenzuela V., Jackson K.L., Sardi S.P., Hetz C. (2018). Gene therapy strategies to restore ER proteostasis in disease. Mol. Ther..

[110] Bard J.A.M., Goodall E.A., Greene E.R., Jonsson E., Dong K.C., Martin A. (2018). Structure and function of the 26S proteasome. Annu. Rev. Biochem..

[111] Gustafsson A.B., Dorn G.W. (2019). Evolving and expanding the roles of mitophagy as a homeostatic and pathogenic process. Physiol. Rev..

[112] Wilkinson S. (2019). ER-phagy: Shaping up and destressing the endoplasmic reticulum. FEBS J..

[113] Glembotski C.C., Arrieta A., Blackwood E.A., Stauffer W.T. (2020). ATF6 as a Nodal Regulator of Proteostasis in the Heart. Front. Physiol..

[114] Maiuolo J., Bulotta S., Verderio C., Benfante R., Borgese N. (2011). Selective activation of the transcription factor ATF6 mediates endoplasmic reticulum proliferation triggered by a membrane protein. Proc. Natl. Acad. Sci. USA.

[115] Jin J.K., Blackwood E.A., Azizi K., Thuerauf D.J., Fahem A.G., Hofmann C., Kaufman R.J., Doroudgar S., Glembotski C.C. (2017). ATF6 decreases myocardial ischemia/reperfusion damage and links ER stress and oxidative stress signaling pathways in the heart. Circ. Res..

[116] Blackwood E.A., Azizi K., Thuerauf D.J., Paxman R.J., Plate L., Kelly J.W., Wiseman R.L., Glembotski C.C. (2019). Pharmacologic ATF6 Activation Confers Global Protection in Widespread Disease Models by Reprograming Cellular Proteostasis. Nat. Commun..

[117] Xu J., Zhou Q., Xu W., Cai L. (2012). Endoplasmic Reticulum Stress and Diabetic Cardiomyopathy. Exp. Diabetes Res..

[118] Li Z., Zhang T., Dai H. (2008). Endoplasmic reticulum stress is involved in myocardial apoptosis of streptozocin-induced diabetic rats. J. Endocrinol..

[119] Kaur N., Raja R., Ruiz-Velasco A., Liu W. (2020). Cellular Protein Quality Control in Diabetic Cardiomyopathy: From Bench to Bedside. Front. Cardiovasc. Med..

[120] Yang L., Zhao D., Ren J., Yang J. (2015). Endoplasmic reticulum stress and protein quality control in diabetic cardiomyopathy. Biochim. Biophys. Acta.

[121] Rutkowski D.T., Hegde R.S. (2010). Regulation of basal cellular physiology by the homeostatic unfolded protein response. J. Cell. Biol..

[122] He Y., Zhou L., Fan Z., Liu S., Fang W. (2018). Palmitic acid, but not highglucose, induced myocardial apoptosis is alleviated by Nacetylcysteine due to attenuated mitochondrial-derived ROS accumulationinduced endoplasmic reticulum stress. Cell Death Dis..

[123] Lopaschuk G.D. (2016). Fatty Acid Oxidation and Its Relation with Insulin Resistance and Associated Disorders. Ann. Nutr. Metab..

[124] Castillero E., Akashi H., Pendrak K., Yerebakan H., Najjar M., Wang C., George I. (2015). Attenuation of the unfolded protein response and endoplasmic reticulum stress after mechanical unloading in dilated cardiomyopathy. Am. J. Physiol. Heart Circ. Physiol..

[125] Yao Y., Lu Q., Hu Z., Yu Y., Chen Q., Wang Q.K. (2017). A non-canonical pathway regulates ER stress signaling and blocks ER stress-induced apoptosis and heart failure. Nat. Commun..

[126] Palomer X., Capdevila-Busquets E., Botteri G., Salvado L., Barroso E., Davidson M.M., Vázquez-Carrera M. (2014). PPARbeta/delta attenuates palmitate-induced endoplasmic reticulum stress and induces autophagic markers in human cardiac cells. Int. J. Cardiol..

[127] Ionita M.G., Arslan F., de Kleijn D.P., Pasterkamp G. (2010). Endogenous inflammatory molecules engage Toll-like receptors in cardiovascular disease. J. Innate Immun..

[128] Salin Raj P., Swapna S.U.S., Raghu K.G. (2019). High glucose induced calcium overload via impairment of SERCA/PLN pathway and mitochondrial dysfunction leads to oxidative stress in H9c2 cells and amelioration with ferulic acid. Fundam. Clin. Pharmacol..

[129] Kumar S., Kain V., Sitasawad S.L. (2012). High glucose-induced Ca2+overload and oxidative stress contribute to apoptosis ofcardiac cells through mitochondrial dependent andindependent pathways. Biochim. Biophys. Acta.

[130] Cai L. (2007). Diabetic cardiomyopathy and its prevention by metallothionein: Experimental evidence, possible mechanisms and clinical implications. Curr. Med. Chem..

[131] Boden G., Cheung P., Kresge K., Homko C., Powers B., Ferrer L. (2014). Insulin resistance is associated with diminished endoplasmic reticulum stress responses in adipose tissue of healthy and diabetic subjects. Diabetes.

[132] Gray S., Kim J.K. (2011). New insights into insulin resistance in the diabetic heart. Trends Endocrinol. Metab..

[133] Tanataweethum N., Zhong F., Trang A., Lee C., Cohen R.N., Bhushan A. (2020). Towards an Insulin Resistant Adipose Model on a Chip. Cell Mol. Bioeng..

[134] Gotoh T., Endo M., Oike Y. (2011). Endoplasmic reticulum stress-related inflammation and cardiovascular diseases. Int. J. Inflamm..

[135] Dos Reis Padilha G., Sanches Machado d’Almeida K., Ronchi Spillere S., Corrêa Souza G. (2018). Dietary Patterns in Secondary Prevention of Heart Failure: A Systematic Review. Nutrients.

[136] Iacobellis G., Barbaro G. (2019). Epicardial adipose tissue feeding and overfeeding the heart. Nutrition.

[137] Yu Y., Wang L., Delguste F., Durand A., Guilbaud A., Rousselin C., Schmidt M.A., Tessier F., Boulanger E., Neviere R. (2017). Advanced glycation end products receptor RAGE controls myocardial dysfunction and oxidative stress in high-fat fed mice by sustaining mitochondrial dynamics and autophagy-lysosome pathway. Radic. Biol. Med..

[138] Hu N., Zhang Y. (2017). TLR4 knockout attenuated high fat dietinduced cardiac dysfunction via NF-κB/JNK-dependent activation of autophagy. Biochim. Biophys. Acta.

[139] Karimi M., Pavlov V.I., Ziegler O., Sriram N., Yoon S.-Y., Agbortoko V., Alexandrova S., Asara J., Sellke F.W., Sturek M. (2019). Robust effect of metabolic syndrome on major metabolic pathways in the myocardium. PLoS ONE.

[140] Cerf M.E. (2018). High Fat Programming and Cardiovascular Disease. Medicina.

[141] Okoshi K., Cezar D.M., Polin A.M., Paladino J.R., Martinez P.F., Oliveira S.A., Lima A.R.R., Damatto R.L., Paiva S.A.R., Zornoff L.A.M. (2019). Influence of intermittent fasting on myocardial infarction-induced cardiac remodeling. BMC Cardiovasc. Disord..

[142] Clifton P.M., Keogh J.B. (2017). A Systematic Review of the Effect of Dietary Saturated and Polyunsaturated Fat on Heart Disease. Nutr. Metab. Cardiovasc. Dis..

[143] Yahfoufi N., Alsadi N., Jambi M., Matar C. (2018). The Immunomodulatory and Anti-Inflammatory Role of Polyphenols. Nutrients.

[144] Cheynier V., Tomas-Barberan F.A., Yoshida K. (2015). Polyphenols: From Plants to a Variety of Food and Nonfood Uses. J. Agric. Food Chem..

[145] Brglez Mojzer E., Knez Hrnčič M., Škerget M., Knez Ž., Bren U. (2016). Polyphenols: Extraction Methods, Antioxidative Action, Bioavailability and Anticarcinogenic Effects. Molecules.

[146] Dudnik A., Gaspar P., Neves A.R., Forster J. (2018). Engineering of Microbial Cell Factories for the Production of Plant Polyphenols with Health-Beneficial Properties. Curr. Pharm. Des..

[147] Gorzynik-Debicka M., Przychodzen P., Cappello F., Kuban-Jankowska A., Marino Gammazza A., Knap N., Wozniak M., Gorska-Ponikowska M. (2018). Potential Health Benefits of Olive Oil and Plant Polyphenols. Int. J. Mol. Sci..

[148] Formisano C., Rigano D., Lopatriello A., Sirignano C., Ramaschi G., Arnoldi L., Knap N., Wozniak M., Gorska-Ponikowska M. (2019). Detailed Phytochemical Characterization of Bergamot Polyphenolic Fraction (BPF) by UPLC-DAD-MS and LC-NMR. J. Agric. Food Chem..

[149] Katarzyna R. (2017). Adult Stem Cell Therapy for Cardiac Repair in Patients After Acute Myocardial Infarction Leading to Ischemic Heart Failure: An Overview of Evidence from the Recent Clinical Trials. Curr. Cardiol. Rev..

[150] Masson J., Liberto E., Beolor J.C., Brevard H., Bicchi C., Rubiolo P. (2016). Oxygenated heterocyclic compounds to differentiate Citrus spp. essential oils through metabolomic strategies. Food Chem..

[151] Parafati M., Lascala A., Morittu V.M., Trimboli F., Rizzuto A., Brunelli E., Coscarelli F., Costa N., Britti D., Ehrlich J. (2015). Bergamot polyphenol fraction prevents nonalcoholic fatty liver disease via stimulation of lipophagy in cafeteria diet-induced rat model of metabolic syndrome. J. Nutr. Biochem..

[152] Musolino V., Gliozzi M., Bombardelli E., Nucera S., Carresi C., Maiuolo J., Mollace R., Paone S., Bosco F., Scarano F. (2020). The synergistic effect of Citrus bergamia and Cynara cardunculus extracts on vascular inflammation and oxidative stress in nonalcoholic fatty liver disease. J. Tradit. Complement. Med..

[153] Mollace V., Rosano G., Anker S., Coats A., Seferovic P., Mollace R., Tavernese A., Gliozzi M., Musolino V., Carresi C. (2021). Pathophysiological Basis for Nutraceutical Supplementation in Heart Failure: A Comprehensive Review. Nutrients.

[154] Gliozzi M., Scarano F., Musolino V., Carresi C., Scicchitano M., Ruga S., Zito M.C., Nucera S., Bosco F., Maiuolo J. (2020). Role of TSPO/VDAC1 Upregulation and Matrix Metalloproteinase-2 Localization in the Dysfunctional Myocardium of Hyperglycaemic Rats. Int. J. Mol. Sci..

[155] Pinti M.V., Fink G.K., Hathaway Q.A., Durr A.J., Kunovac AHollander J.M. (2019). Mitochondrial dysfunction in type 2 diabetes mellitus: An organ-based analysis. Am. J. Physiol. Endocrinol. Metab..

[156] Lopez-Crisosto C., Pennanen C., Vásquez-Trincado C., Morales P.E., Bravo-Sagua R., Quest A.F.G., Chiong M., Lavandero S. (2017). Sarcoplasmic reticulum-mitochondria communication in cardiovascular pathophysiology. Nat. Rev. Cardiol..

[157] Zhang N., Yang Z., Xiang S.Z., Jin Y.G., Wei W.Y., Bian Z.Y., Deng W., Tang Q.Z. (2016). Nobiletin attenuates cardiac dysfunction, oxidative stress, and inflammatory in streptozotocin: Induced diabetic cardiomyopathy. Mol. Cell Biochem..

[158] Mahmoud A.M., Bautista R.J.H., Sandhu M.A., Hussein O.E. (2019). Beneficial Effects of Citrus Flavonoids on Cardiovascular and Metabolic Health. Oxidative Med. Cell. Longev..

[159] Mallick N., Khan R.A. (2016). Antihyperlipidemic effects of Citrus sinensis, Citrus paradisi, and their combinations. J. Pharm Bioall Sci..

[160] Di Donna L., De Luca G., Mazzotti F., Napoli A., Salerno R., Taverna D., Sindona G. (2009). Statin-like principles of bergamot fruit (Citrus bergamia): Isolation of 3-hydroxymethylglutaryl flavonoid glycosides. J. Nat. Prod..

[161] Parim B., Sathibabu Uddandrao V.V., Saravanan G. (2019). Diabetic cardiomyopathy: Molecular mechanisms, detrimental effects of conventional treatment, and beneficial effects of natural therapy. Heart Fail. Rev..

[162] Chtourou Y., Aouey B., Aroui S., Kebieche M., Fetoui H. (2016). Anti-apoptotic and anti-inflammatory effects of naringin on cisplatin-induced renal injury in the rat. Chem.-Biol. Int..

[163] Chen R., Qi Q.-L., Wang M.-T., Li Q.-Y. (2016). Therapeutic potential of naringin: An overview. Pharm. Biol..

[164] Zhao B.T., Kim E.J., Son K.H., Son J.K., Min B.S., Woo M.H. (2015). Quality evaluation and pattern recognition analyses of marker compounds from five medicinal drugs of Rutaceae family by HPLC/PDA. Arch. Pharm. Res..

[165] Seo C.S., Shin H.K. (2020). Quality assessment of traditional herbal formula, Hyeonggaeyeongyotang through simultaneous determination of twenty marker components by HPLC-PDA and LC-MS/MS. Saudi Pharm. J..

[166] Wu J., Huang G., Li Y., Li X. (2020). Flavonoids from *Aurantii Fructus Immaturus* and *Aurantii Fructus*: Promising phytomedicines for the treatment of liver diseases. Chin. Med..

[167] Varshney V., Garabadu D. (2021). Naringin Exhibits Mas Receptor-Mediated Neuroprotection Against Amyloid Beta-Induced Cognitive Deficits and Mitochondrial Toxicity in Rat Brain. Neurotox Res..

[168] Zhang Y.F., Meng N.N., Li H.Z., Wen Y.J., Liu J.T., Zhang C.L., Yuan X.H., Jin X.D. (2018). Effect of naringin on oxidative stress and endoplasmic reticulum stress in diabetic cardiomyopathy. Zhongguo Zhong Yao Za Zhi.

[169] Shangguan W.J., Zhang Y.H., Li Z.C., Tang L.M., Shao J., Li H. (2017). Naringin inhibits vascular endothelial cell apoptosis via endoplasmic reticulum stress- and mitochondrial-mediated pathways and promotes intraosseous angiogenesis in ovariectomized rats. Int. J. Mol. Med..

[170] Werner S.L., Barken D., Hoffmann A. (2005). Stimulus specificity of gene expression programs determined by temporal control of IKK activity. Science.

[171] Qiong Y., Zijun W., Bin W., Chang L., Ruina H., Li Y. (2016). Naringin protects cardiomyocytes against hyperglycemia-induced injuries in vitro and in vivo. J. Endocrinol..

[172] Fu D., Mui D., Zhu H., Zhang Y. (2020). Exenatide inhibits NF-κB and attenuates ER stress in diabetic cardiomyocyte models. Aging (Albany NY).

[173] Cassidy A., Minihane A.M. (2017). The role of metabolism (and the microbiome) in defining the clinical efficacy of dietary flavonoids. Am. J. Clin. Nutr..

[174] Truzzi F., Tibaldi C., Zhang Y., Dinelli G.D., Amen E. (2021). An Overview on Dietary Polyphenols and Their Biopharmaceutical Classification System (BCS). Int. J. Mol. Sci..

[175] Spigoni V., Mena P., Fantuzzi F., Tassotti M., Brighenti F., Bonadonna R.C., Del Rio D., Dei Cas A. (2017). Bioavailability of Bergamot (Citrus bergamia) Flavanones and Biological Activity of Their Circulating Metabolites in Human Pro-Angiogenic Cells. Nutrients.

[176] Ávila-Gálvez M.A., Giménez-Bastida J.A., González-Sarrías A., Espín J.C. (2021). New Insights into the Metabolism of the Flavanones Eriocitrin and Hesperidin: A Comparative Human Pharmacokinetic Study. Antioxidants.

[177] Chanet A., Milenkovic D., Claude S., Maier J.A.M., Kamran M., Rakotomanomana K.N., Shinkaruk S., Be´rard A.M., Bennetau-Pelissero C., Mazur A. (2013). Flavanone metabolites decrease monocyte adhesion to TNF-*a*-activated endothelial cells by modulating expression of atherosclerosis-related genes. Br. J. Nutr..

[178] Giménez-Bastida J.A., González-Sarrías A., Vallejo F., Espín J.C., Tomás-Barberán F. (2016). Hesperetin and its sulfate and glucuronide metabolites inhibit TNF-α induced human aortic endothelial cell migration and decrease plasminogen activator inhibitor-1 (PAI-1) levels. Food Funct.

[179] Gao J., Shi X., He H., Zhang J., Lin D., Fu G., Lai D. (2017). Assessment of Sarcoplasmic Reticulum Calcium Reserve and Intracellular Diastolic Calcium Removal in Isolated Ventricular Cardiomyocytes. J. Vis. Exp..

[180] Mandavia C.H., Aroor A.R., Demarco V.G., Sowers J.R. (2013). Molecular and metabolic mechanisms of cardiac dysfunction in diabetes. Life Sci..

[181] Jia G., Hill M.A., Sowers J.R. (2018). Diabetic Cardiomyopathy: An Update of Mechanisms Contributing to This Clinical Entity. Circ. Res..

[182] Tong M., Saito T., Zhai P., Oka S.I., Mizushima W., Nakamura (2019). Mitophagy Is Essential for Maintaining Cardiac Function During High Fat Diet-Induced Diabetic Cardiomyopathy. Circ. Res..

[183] Wu L., Wang K., Wang W., Wen Z., Wang P., Liu L., Wang D.W. (2018). Glucagon-like peptide-1 ameliorates cardiac lipotoxicity in diabetic cardiomyopathy via the PPARalpha pathway. Aging Cell..

[184] Bodiga V.L., Eda S.R., Bodiga S. (2014). Advanced glycation end products: Role in pathology of diabetic cardiomyopathy. Heart Fail. Rev..

[185] Borghetti G., von Lewinski D., Eaton D.M., Sourij H., Houser S.R., Wallner M. (2018). Diabetic Cardiomyopathy: Current and Future Therapies. Beyond Glycemic Control. Front. Physiol..

[186] Arauna D., Furrianca M., Espinosa-Parrilla Y., Fuentes E., Alarcón M., Palomo I. (2019). Natural Bioactive Compounds as Protectors of Mitochondrial Dysfunction in Cardiovascular Diseases and Aging. Molecules.

[187] Sharifi-Rad J., Rodrigues C.F., Sharopov F., Docea A.O., Can Karacam A., Sharifi-Rad M., Karıncaoglu D.K., Gülseren G., Şenol E., Demircan E. (2020). Diet, Lifestyle and Cardiovascular Diseases: Linking Pathophysiology to Cardioprotective Effects of Natural Bioactive Compounds. Int. J. Environ. Res. Public Health.

[188] Navarra M., Mannucci C., Delbò M., Calapai G. (2015). Citrus bergamia essential oil: From basic research to clinical application. Front. Pharmacol..

[189] Toth P.P., Patti A.M., Nikolic D., Giglio R.V., Castellino G., Biancucci T., Rizzo M. (2015). Bergamot reduces plasma lipids, atherogenic small dense LDL, and subclinical atherosclerosis in subjects with moderate hypercholesterolemia: A 6 months prospective study. Front. Pharmacol..

[190] Bruno A., Pandolfo G., Crucitti M., Maisano A., Zoccali R.A., Muscatello M.R.A. (2017). Metabolic outcomes of bergamot polyphenolic fraction administration in patients treated with second-generation antipsychotics: A pilot study. J. Nutr. Biochem..

[191] Bhia M., Motallebi M., Abadi B., Zarepour A., Pereira-Silva M., Saremnejad F., Santos A., Zarrabi A., Melero A., Jafari S. (2021). Naringenin Nano-Delivery Systems and Their Therapeutic Applications. Pharmaceutics.

[192] Cheng Y.C., Sheen J.M., Hu W.L., Hung Y.C. (2017). Polyphenols and Oxidative Stress in Atherosclerosis-Related Ischemic Heart Disease and Stroke. Oxid. Med. Cell Longev..

[193] Ilkun O., Boudina S. (2013). Cardiac dysfunction and oxidative stress in the metabolic syndrome: An update on antioxidant therapies. Curr. Pharm. Des..

